# High Prevalence of Sexually Transmitted Infections and Poor Sensitivity and Specificity of Screening Algorithms for Chlamydia and Gonorrhea Among Female Sex Workers in Zimbabwe: Analysis of Respondent-Driven Sampling Surveys in 3 Communities

**DOI:** 10.1097/OLQ.0000000000002086

**Published:** 2025-01-08

**Authors:** Sungai T. Chabata, Elizabeth Fearon, Sithembile Musemburi, Fortunate Machingura, Anna Machiha, James R. Hargreaves, Francis J. Ndowa, Owen Mugurungi, Frances M. Cowan, Richard Steen

**Affiliations:** From the ∗ Centre for Sexual Health and HIV/AIDS Research (CeSHHAR) Zimbabwe, Harare, Zimbabwe; † Department of Public Health, Erasmus MC, University Medical Center Rotterdam, Rotterdam, the Netherlands; ‡ Department of Global Health and Development, London School of Hygiene and Tropical Medicine; § Institute for Global Health, University College London, London; ¶ Department of International Public Health, Liverpool School of Tropical Medicine, Liverpool, United Kingdom; ∥ Ministry of Health and Child Care Zimbabwe, Harare, Zimbabwe; ∗∗ Centre for Evaluation, London School of Hygiene and Tropical Medicine, London, United Kingdom; †† Skin & Genito-Urinary Medicine Clinic, Harare, Zimbabwe

## Abstract

**Background:**

Effective strategies to reduce sexually transmitted infection burden and transmission among female sex workers (FSWs) and their networks are needed. We report sexually transmitted infection prevalence among FSWs in Zimbabwe and investigate the performance of screening algorithms.

**Methods:**

Respondent-driven sampling (RDS) surveys, including blood sampling for syphilis serology, were conducted among FSWs in 3 communities in Zimbabwe in 2017. In addition, a random sample of one-third of participants were offered genital examination and sexually transmitted infection (STI) testing. Data on symptoms and clinical signs were analyzed to determine the proportion of asymptomatic and clinically inapparent STIs by HIV status, and the sensitivity, specificity, and predictive values of syndromic, clinical, and hybrid screening algorithms for chlamydia and gonorrhea. Analyses were RDS-II weighted.

**Results:**

Overall, 2507 women were included in the RDS surveys, and 661 of 836 (79.1%) of those randomly offered genital examination and STI testing accepted. The prevalence of STI by site ranged from 15.7% to 20.0% for syphilis (rapid plasma reagin + *Treponema pallidum* hemagglutination assay), 6.8% to 14.3% for gonorrhea, 8.4% to 10.1% for chlamydia, 26.6% to 35.5% for trichomonas, and 37.0% to 47.6% for any high-risk human papilloma virus. A high proportion of infections were both asymptomatic and clinically undetectable (gonorrhea: 41.2%, chlamydia: 51.7%, trichomonas: 62.8%). Screening algorithms performed poorly whether based on symptoms only (sensitivity: 53.3% gonorrhea, 43.3% chlamydia) or either symptoms or clinical signs (sensitivity: 58.7% gonorrhea, 48.3% chlamydia).

**Conclusions:**

Sexually transmitted infection burden is high among FSWs in Zimbabwe. The low sensitivity and specificity of screening algorithms used to guide syndromic management mean that more effective approaches are required to strengthen STI control. As access to HIV-specific prevention methods like preexposure prophylaxis increases, support for consistent condom use needs to be strengthened.

Globally, an estimated 1 million curable sexually transmitted infections (STIs) are acquired every day. The World Health Organization estimated that there were 376.4 million new cases of chlamydia, gonorrhea, syphilis, and trichomoniasis among people 15 to 49 years old, comprising 127.2 million cases of chlamydia, 86.9 million cases of gonorrhea, 156 million cases of trichomoniasis, and 6.3 million cases of syphilis.^1^

The World Health Organization Global Health Sector Strategy on STIs, 2022–2030, aims to eliminate STIs as a public health threat by 2030.^2^ Evidence from Asia and elsewhere supports the feasibility of this aim if sex workers and other key populations are reached with effective interventions that support consistent condom use and effective STI screening and treatment.^3^ Especially because there is evidence that increasing usage of HIV-specific prevention methods such as preexposure prophylaxis (PrEP) is reducing condom use, further destabilizing STI control.^4^

Sexually transmitted infections are a recognized occupational hazard for sex workers due to high prevalence and frequent exposure. Untreated, an estimated 8% to 20% of women with gonorrhea or chlamydia suffer serious sequelae including pelvic inflammatory disease.^5^ Pelvic inflammatory disease contributes to increased maternal mortality due to post-abortion and postpartum infections and sepsis, and a 6 to 10 times higher risk of ectopic pregnancy.^4^ Risk of infertility has been estimated at 15% to 25% after one episode of pelvic inflammatory disease and 50% to 60% after a third episode, likely even higher where antibiotic treatment is not readily available.^4^ Among pregnant women with untreated syphilis, two-thirds of pregnancies are lost or newborns born with congenital syphilis.^6^ Male and transgender sex workers also suffer a high burden of often asymptomatic STIs with a range of potential complications.^7^

Sexually transmitted infections were poorly controlled among Zimbabwean sex workers when HIV emerged in the late 1980s. Incidence declines were documented during the 1990s as condom access improved and peer-based interventions succeeded in raising condom use in sex work. Between 1992 and 1996, STI syndromes reduced by 60% in Harare, and countrywide by a third between 1989 and 1999.^8^ By the early 2000s, transmission of both HIV and other STIs within and beyond sex work networks seems to have reached a low point.^9^ However, sex work subsequently rebounded as economic activity picked up again after 2010.^10^ Recent studies of STI etiologies among symptomatic women and men seeking care reveal high STI prevalence, even beyond sex work networks. Among 200 women presenting to 6 primary care clinics with abnormal vaginal discharge in 2014 to 2015, 24% were found to have gonorrhea and 14% chlamydia.^11^

Zimbabwe has experienced some of the highest rates of cervical cancer in the world.^12^ Since 2018, bivalent human papillomavirus (HPV) genotype 16 and 18 vaccination has been offered to girls aged 10 to 14 years.^13^ Data on prevalence of other high-risk HPV genotypes are needed to inform prevention and vaccination strategies.

High prevalence or a large proportion of asymptomatic infections and frequent, often serious complications make screening a priority for populations at highest risk, including sex workers. However, in the absence of available testing, national STI treatment guidelines in Zimbabwe, as in many countries, follow syndromic algorithms, which are only used when patients present with symptoms. Experience from Zimbabwe's nationally scaled program for sex workers (Key Populations [KP] program, detailed elsewhere^14^), where female sex workers (FSWs) are recommended to visit the clinic every 3 months even without presenting any symptoms, suggests that clinical signs found on routine examination could potentially be useful in screening for asymptomatic STIs such as gonorrhea and chlamydia.

This study reports STI prevalence in Zimbabwe and explores the limitations of screening methods based on clinical signs and symptoms. We (1) assessed the prevalence of syphilis, gonorrhea, chlamydia, trichomonas, and HPV by HIV status among FSWs residing in Harare, Bulawayo, and a smaller rural site in Zimbabwe, including different high-risk HPV genotypes for cervical cancer, and (2) calculated the sensitivity, specificity, and predictive values of several screening algorithms for chlamydia and gonorrhea based on common symptoms and clinical signs.

## MATERIALS AND METHODS

### Population and Setting

This study was nested within a population size estimation study among FSWs residents in Zimbabwe's 2 largest cities of Harare and Bulawayo and a smaller rural mining town, which has been reported on elsewhere.^15^ Eligibility included being a woman 18 years or older, having exchanged sex for money or gifts in the previous 30 days, and having lived or worked at the study site for at least the previous 30 days. Female sex workers only making temporary visits of under 1 month to the study site were excluded.

### Data Collection

#### Respondent-Driven Sampling Surveys

Respondent-driven sampling (RDS) is a network-based sampling technique that leverages social relationships to recruit and obtain population representative estimates from populations lacking sampling frames^16^ and has been extensively used in the recruitment of FSWs around the world including in Zimbabwe. Seed participants are selected and given coupons with which to recruit from their social networks of the target population; subsequent recruited participants are given coupons themselves for onward sample “wave” recruitment, and this process continues until the desired sample size is reached.^16^ A statistical framework that estimates a sampling probability and weights for each participant is then used to make inferences about the broader network of the target population.

Between March and April 2017, FSWs were recruited in separate RDS surveys in each site with 20, 12, and 6 initial “seed” participants in Harare, Bulawayo, and Shamva, respectively. Formative research was conducted to profile and purposively select “seed” participants from common FSW hotspots such as truck stops, bars, night clubs and streets, and other less common hotspots such as markets and homes. The purposive sample of “seed” participants represented the diversity of sex work typologies, geographical areas, and ages that were identified during the rapid ethnographic mapping before RDS surveys.^17^

At the RDS survey station, each recruited participant was screened for eligibility by a survey assistant, provided written informed consent, and completed an interviewer-administered questionnaire. A research nurse collected a finger-prick blood sample for HIV antibody testing using national testing algorithms and syphilis testing protocols. For HIV testing, Determine HIV-1/2 or First Response HIV-1-2 kit antibody testing was used as the first screening test. Where the result was HIV positive, this was confirmed using First Response HIV-1-2 kit or Determine HIV-1/2. Where the 2 test results were discordant, repeat testing was advised within 2 weeks. One in 3 nonseed participants were randomly selected for genital examination and screening for STIs. All laboratory results were returned to participants within 2 weeks, and there was a follow-up mechanism in place through the program to refer those who tested positive to the nearest clinic for treatment.

Following completion of survey procedures, a survey assistant issued each participant 2 coupons and asked them to refer 2 peers (recruits). Iteratively, each recruit was given 2 coupons to refer a further 2 peers. Participants received $5 for completing the questionnaire and HIV/syphilis testing. Participants received an additional $2 for each recruit they successfully referred (up to a maximum of 2).

### Sample Size

Sample size calculations were based primarily on requirements for population size estimation at each site,^15^ and secondarily on estimating HIV prevalence with adequate precision accounting for the RDS sampling design.^18^ Our targeted sizes were 1500 FSWs in Harare, 800 FSWs in Bulawayo, and 200 FSWs in Shamva.

### STI Laboratory Data and Genital Examinations

Samples were tested for HIV according to the Zimbabwe National HIV testing algorithm in series.^19^ The syphilis sample was tested using Chembio DPP Syphilis Screen & Confirm Assay (Chembio Diagnostic Systems, Medford, NY), and results were given to participants. All participants testing syphilis positive were asked to provide an additional blood sample to run a Confirm POC test, a near-patient assay that tests for both rapid plasma reagin (RPR) and *Treponema pallidum* hemagglutination assay (TPHA).

One in 3 randomly selected participants was invited for an STI study: STI testing for *Neisseria gonorrhoeae* (NG), *Chlamydia trachomatis* (CT), *Trichomonas vaginalis* (TV), high-risk and oncogenic HPV, genital examination, and questions about symptoms of STIs. Participants were asked about symptoms of STIs before they underwent a genital examination. Signs of STI were recorded. Examined participants had a vaginal swab collected for TV using Xpert TV (Cepheid) plus cervical swabs collected to test for NG and CT using Xpert CT/NG (Cepheid). Participants also had a cervical sample collected using a cervix brush and placed in PreservCyt 20-mL vial solution (Hologic, Marlborough, MA) for Xpert HPV. All STI samples were transported to the Flow Cytometry Laboratory in Harare within 48 hours at 4°C.

### Ethics Approval

Ethics approval was obtained from the Medical Research Council of Zimbabwe, University College London, the London School of Hygiene & Tropical Medicine, and RTI International. Written informed consent was obtained from participants before enrollment.

### Measures

Questionnaire data included sociodemographic and economic characteristics of sex work; sexual behavior; psychological health; physical health; history of STI symptoms and actions taken as a result; sexual and social networks; social capital; use of services, including HIV testing, antiretroviral therapy, prevention of mother-to-child transmission, and family planning; history of gender-based violence; and psychological health.

We assessed several screening algorithms adapted from the syndromic case management algorithm for vaginal discharge, recommended for use as a screening tool by the Ministry of Health and Child Care in Zimbabwe in 2018 (online Supplemental Appendix Fig. 2, http://links.lww.com/OLQ/B143). Sensitivities, specificities, and predictive values of symptoms and clinical signs for detecting NG and CT were calculated without including risk assessment for cervicitis (nearly always positive for FSWs). Clinical signs from speculum examination were included and assessed as a screening algorithm (with or without step 1 symptoms as entry point). A hybrid screening algorithm based on presence of any of the aforementioned symptoms or signs was also evaluated.

### Statistical Analysis

We weighted our analyses of the characteristics of FSWs and HIV and STI prevalence separately for each site, using data from the RDS surveys and RDS-II weighting, dropping seed participants. The RDS-II estimator weights participants' responses by the inverse of their reported personal network size, which we determined by asking women how many other FSWs they knew who resided at the site, were older than 18 years, whom they had seen in the past month, and to whom they would consider giving a coupon to participate in the study. Knowing was defined as “you know their name and they know yours.”

Among the one-third of women who were randomized to be offered STI testing and genital examination, we used RDS-II weighting for STI prevalence estimates. Although participants were randomly invited for examination and testing, not all accepted. To assess the possibility of selection bias, we compared those who did and did not take up the offer with respect to site, sociodemographic characteristics, sex work characteristics, HIV status, and prevalence of self-reported symptoms of STIs used *χ*^2^ tests and *t* tests.

We then used laboratory STI data to assess the sensitivity, specificity, and positive and negative predictive values of 3 screening algorithms using (1) self-reported vaginal symptoms only, (2) signs upon genital examination only, and (3) symptoms or signs as above (hybrid algorithm), for identifying NG and CT. Signs of NG and CT included abnormal vaginal discharge or any one of cervical mucopus, cervical friability, or cervical excitation tenderness.

We used the RDS package for R statistical software^20^ version 3.3.2 and STATA 17.

## RESULTS

### Sociodemographic Characteristics of Participants in the RDS Surveys

There were 1497 FSWs recruited in Harare (March–April 2017), 808 in Bulawayo (April–May 2017), and 202 in Shamva (April–May 2017), with 6 waves of recruitment in Bulawayo and Shamva and 7 waves in Harare. Compared with FSWs who accepted STI testing, those who consented to the RDS survey and were randomized but refused STI testing and examination were more likely to be pregnant, younger, more likely to be never married, less likely to be aware of status if HIV positive, and less likely to be virally suppressed, although there was no evidence for a difference in HIV prevalence (online Supplemental Appendix Table 2, http://links.lww.com/OLQ/B143). There was little evidence that refusers differed by site, reported condom use with clients, or STI symptoms reported in the RDS survey.

Across the 3 sites, 23% to 29% of women were younger than 25 years, 53% to 61% had started sex work before the age of 25 years, and 21% to 35% reported more than 10 clients per week. Mobility was also high with 36% to 44% having worked in other sites during past year (Table 1). The proportion of FSWs who attended the KP program in the past 6 months was higher in Harare (22.9%) and Bulawayo (25.6%) compared with Shamva (1.2%).

**TABLE 1:** Sociodemographic and Sex Work Characteristics of FSWs Recruited Into RDS Surveys by Site

	Harare (N = 1497)		Bulawayo (N = 808)		Shamva (N = 202)	
	n	% (RDS-II Weighted)	n	% (RDS-II Weighted)	n	% (RDS-II Weighted)
Age at survey, y						
18–19	41	3.3	64	8.9	6	3.7
20–24	291	19.6	155	20.1	38	22.9
≥25	1165	77.1	589	71.0	158	73.4
Education						
None	28	1.8	7	0.8	7	3.7
Primary	495	35.1	211	27.1	82	45.2
Secondary	953	61.7	568	68.8	112	50.9
Tertiary	20	1.4	22	3.3	1	0.2
Marital status						
Never married	247	15.2	289	37.0	10	3.3
Divorced/Separated	1026	70.4	394	48.1	163	79.2
Widowed	217	13.8	108	13.1	29	17.5
Currently married	7	0.7	17	1.8	0	0.0
Age started sex work, y						
<18	170	11	132	17	18	12.6
18–19	177	12.1	102	13.1	19	12.2
20–24	451	30.3	244	30.9	71	29.2
≥25	699	46.6	330	39.1	94	46
Duration in sex work, y						
≤2	364	27.1	225	33.9	57	29.2
>2	1133	72.9	583	66.1	145	70.8
No. clients in the previous week						
0	49	3.6	38	6.3	11	8.9
1–5	406	32.3	368	48.5	73	37.8
6–10	422	28.8	214	24.5	45	21.8
10–15	209	12.4	78	7.6	30	13
16+	411	22.9	110	13	43	18.5
Worked in other sites as a sex worker in last 12 mo						
No	949	63.7	473	63.5	112	56.1
Yes	548	36.3	335	36.5	90	43.9
Attended the KP program in past 6 mo						
No	1122	77.1	566	74.4	199	98.8
Yes	375	22.9	239	25.6	3	1.2

### HIV and STI Prevalence Among FSWs Participating in the Study

Overall, HIV prevalence was consistently high across all sites, ranging from 52.3% to 54.8% (Table 2). For the STI substudy, 836 FSWs were invited and 661 (79%) accepted. The prevalence of STIs varied across sites (Table 2) from 15.7% to 20.0% for syphilis (TPHA + RPR positive), 6.8% to 14.3% for gonorrhea, 8.4% to 10.1% for chlamydia, 26.6% to 35.5% for TV, and 37.0% to 47.6% for HPV (any high-risk [HR-HPV]). HR-HPV genotypes were varied, with a type 16 estimated prevalence of 9.1% to 15.5% across sites, type 18/45 prevalence of 9.4% to 17.2%; and another 30.6% to 39.7% prevalence of other HR-HPV genotypes.

**TABLE 2:** HIV And STI Prevalence Among FSWs Recruited Into RDS Surveys by Site, RDS-II Weighted

	Harare (N = 1497)		Bulawayo (N = 808)		Shamva (N = 202)	
	n/N	% (95% CI)	n/N	% (95% CI)	n/N	% (95% CI)
HIV	822/1482	54.5 (51.0–57.8)	449/804	52.3 (47.7–56.8)	111/200	54.8 (45.4–63.9)
Syphilis: RPR and TPHA positive	227/1497	15.7 (11.9–19.6)	159/808	20.0 (14.4–25.5)	39/202	19.9 (11.7–28.1)
Syphilis: RPR negative and TPHA positive	25/1497	1.7 (1.1–2.7)	25/808	2.7 (1.6–4.6)	7/202	2.6 (1.0–6.8)
*Neisseria gonorrhoeae* (NG)	47/406	10.2 (4.7–15.7)	16/210	6.8 (0.9–14.6)	14/58	14.3 (2.6–26.0)
*Chlamydia trachomatis* (CT)	38/406	8.4 (3.1–13.7)	17/210	8.7 (3.6–21.0)	6/58	10.1 (2.6–17.5)
*Trichomoniasis vaginalis* (TV)	114/406	27.6 (18.9–36.4)	65/210	30.5 (18.5–42.5)	19/58	26.6 (7.9–45.3)
Any STI (NG, CT, TV, syphilis: RPR and TPHA positive)	193/406	49.1 (42.5–55.8)	103/210	50.2 (41.3–59.1)	35/58	47.4 (29.9–65.7)
HPV type 16	53/406	13.2 (7.5–18.9)	24/210	9.3 (0.4–19.1)	9/58	9.2 (0.3–18.8)
HPV type 18/45	41/406	9.4 (2.5–16.3)	22/210	11.0 (2.1–19.9)	10/58	9.3 (1.1–17.5)
Other high-risk HPV	157/406	39.5 (30.3–48.6)	68/210	30.6 (19.3–42.0)	23/58	35.7 (14.2–57.2)
Any of the above high-risk HPV	190/406	47.6 (37.9–57.3)	85/210	37.0 (24.6–49.5)	30/58	42.2 (18.8–65.6)

CI indicates confidence interval.

The prevalence of syphilis was higher among HIV-positive FSWs in Harare (20.1% vs. 11.0%) and Bulawayo (27.6% vs. 11.2%) compared with HIV-negative FSWs, whereas it was similar in both groups in Shamva (19.4% vs. 19.5%; Table 3). The proportion of FSWs testing positive for *N. gonorrhoeae* was comparable regardless of HIV status in Harare (9.6% vs. 11.7%) and Bulawayo (5.8% vs. 8.0%) but was higher among HIV-negative individuals compared with HIV-positive ones in Shamva (19.3% vs. 10.6%). The proportion of FSWs testing positive for *C. trachomatis*, HPV type 16, other high-risk HPV, or any high-risk HPV was higher among HIV-negative FSWs compared with HIV-positive FSWs across all sites.

**TABLE 3:** STI Prevalence Among FSWs Recruited Into RDS Surveys by HIV Status by Site, RDS-II Weighted

STIs: Laboratory Tested	Harare (N = 1497)				Bulawayo (N = 808)				Shamva (N = 202)			
	HIV-Negative(N = 660)		HIV-Positive(N = 822)		HIV-Negative(N = 355)		HIV-Positive(N = 449)		HIV-Negative(N = 89)		HIV-Positive(N = 111)	
	n/N	% (95% CI)	n/N	% (95% CI)	n/N	% (95% CI)	n/N	% (95% CI)	n/N	% (95% CI)	n/N	% (95% CI)
Syphilis: RPR and TPHA positive	65/660	11.0 (7.9–15.1)	161/822	20.1 (16.7–24.1)	32/355	11.2 (7.4–16.6)	125/449	27.6 (22.3–33.6)	15/89	19.5 (10.8–32.8)	24/111	19.4 (11.0–31.8)
Syphilis: RPR negative and TPHA positive	8/660	1.2 (0.5–2.9)	17/822	2.1 (1.2–3.8)	7/355	1.6 (0.7–3.6)	18/449	3.8 (2.0–7.2)	5/89	4.2 (1.3–12.9)	2/111	1.3 (0.2–7.1)
*Neisseria gonorrhoeae* (NG)	19/173	11.7 (6.7–19.4)	27/231	9.6 (5.9–15.1)	6/87	8.0 (3.2–18.3)	10/121	5.8 (2.4–13.2)	6/25	19.3 (6.6–44.9)	8/32	10.6 (3.7–27.0)
*Chlamydia trachomatis* (CT)	25/173	13.8 (8.5–21.5)	13/231	4.9 (2.4–9.5)	9/87	13.1 (5.6–27.7)	8/121	4.7 (2.1–10.2)	4/25	20.1 (6.7–46.8)	2/32	2.6 (0.5–13.4)
*Trichomoniasis vaginalis* (TV)	43/173	26.6 (18.6–36.5)	70/231	28.3 (20.8–37.1)	17/87	18.5 (10.4–30.7)	47/121	40.1 (28.9–52.4)	5/25	15.7 (4.9–40.1)	13/32	34.5 (14.5–62.0)
Any STI (NG, CT, TV, syphilis: RPR and TPHA positive)	74/173	50.1 (40.3–59.8)	118/231	48.6 (39.6–57.6)	30/87	36.1 (24.0–50.3)	71/121	62.1 (49.9–72.9)	14/25	47.5 (23.2–73.0)	20/32	47.2 (22.5–73.4)
HPV type 16	14/173	6.9 (3.4–13.5)	39/231	17.3 (11.1–25.9)	5/87	6.4 (2.1–17.4)	19/121	12.0 (6.5–21.0)	3/25	4.9 (1.2–18.0)	6/32	12.5 (4.1–32.3)
HPV type 18/45	10/173	4.6 (2.1–9.9)	30/231	12.2 (7.4–19.4)	4/87	4.7 (1.5–14.3)	18/121	16.6 (9.7–26.9)	4/25	14.1 (4.3–37.4)	6/32	5.8 (1.9–16.0)
Other high-risk HPV	53/173	28.2 (20.7–37.2)	104/231	47.4 (38.4–56.6)	18/87	17.4 (9.8–29.1)	50/121	43.6 (32.1–55.9)	7/25	22.3 (8.3–47.4)	16/32	45.8 (21.7–72.1)
Any of the above high-risk HPV	63/173	32.5 (24.5–41.8)	126/231	57.7 (48.8–66.2)	23/87	23.1 (14.1–35.5)	62/121	50.6 (38.6–62.6)	11/25	32.4 (14.4–57.7)	19/32	49.6 (24.0–75.5)

CI indicates confidence interval.

Gonorrhea and chlamydia infections correlated poorly with symptoms and clinical signs (Table 4). Sensitivity of the screening algorithm (Table 4A, symptoms only, assuming all FSW scores were risk-positive) was 53% for gonorrhea and 43% for chlamydia. Sensitivity dropped to 32% (NG) and 30% (CT) in a screening algorithm where only clinical signs were used (Table 4B) but increased to 59% (NG) and 48% (CT) in a hybrid screening algorithm where treatment is given if either symptoms or clinical signs are present (Table 4B). Specificities and positive predictive values were low in all cases, whereas negative predictive values were high, >90%.

**TABLE 4:** Validation of Screening Algorithms for NG/CT Based on Symptoms, Signs, or Combination

(A) Sensitivity, Specificity, PPV, and NPV Based on Self-Reported Vaginal Itching, Burning, and/or Discharge Only				
STI	Sensitivity	Specificity	PPV	NPV
	n/N (%)	n/N (%)	n/N (%)	n/N (%)
Chlamydia	26/60 (43.3)	408/594 (68.7)	26/212 (12.3)	408/442 (92.3)
Gonorrhea	40/75 (53.3)	407/579 (70.3)	40/212 (18.9)	407/442 (92.1)

(B) Sensitivity, Specificity, PPV, and NPV Based on Clinical Signs Upon Examination (Abnormal Discharge, Cervical Signs), No Regard to Self-Reported Symptoms				
STI	Sensitivity	Specificity	PPV	NPV
	n/N (%)	n/N (%)	n/N (%)	n/N (%)
Chlamydia	18/60 (30.0)	493/594 (83.0)	18/119 (15.1)	493/535 (92.1)
Gonorrhea	24/75 (32.0)	484/579 (83.6)	24/119 (20.2)	484/535 (90.5)

(C) Sensitivity, Specificity, PPV, and NPV Based on EITHER Clinical Signs Upon Examination (Abnormal Discharge, Cervical Signs) OR Self-Reported Symptoms				
STI	Sensitivity	Specificity	PPV	NPV
	n/N (%)	n/N (%)	n/N (%)	n/N (%)
Chlamydia	29/60 (48.3)	371/594 (62.5)	29/252 (11.5)	371/402 (92.5)
Gonorrhea	44/75 (58.7)	371/579 (64.1)	44/252 (17.5)	371/402 (92.5)

NPV indicates negative predictive value; PPV, negative predictive value.

Using symptoms (vaginal itching, burning, and/or discharge) as entry point for the algorithm (Table 4A) missed half of all NG and/or CT infections. Prevalences were 16% (NG) and 11% (CT) among those with symptoms, but 9% (NG) and 8% (CT) among those without symptoms.

## DISCUSSION

Sexually transmitted infections remain common and important occupational hazards for Zimbabwean FSWs. Among FSWs who were selected to participate in the STI study, 47% to 50% tested positive for any STI, whereas 37% to 48% tested positive for any high-risk HPV, across the 3 sites. Gonorrhea and chlamydia infections correlated poorly with symptoms and clinical signs. High prevalence and frequent exposure create a heavy burden of serious morbidity for FSWs themselves and help sustain STI transmission within and beyond sex work networks.

We found a higher prevalence of chlamydia (8.4%–10.1% across sites) and gonorrhea (6.8%–14.3%) compared with estimates from a systematic review, which summarized genital prevalence among FSWs across Sub-Saharan Africa at 4.2% to 7.3% and 5.4% to 11.0% respectively.^21^ Incidence and prevalence of bacterial STIs have also been found to be high in intervention studies among young women attending sexual and reproductive health services^22^ and among those eligible for PrEP^23^ in Zimbabwe, who have not been recruited on the basis of sex work. Prevalence of active syphilis in a population representative survey of women aged 15+ years was estimated in 2015/2016 to be 1%, as expected lower than we found among FSWs; as in this survey, we also observed higher active syphilis prevalence among FSWs living with HIV compared with those HIV negative.^24^

Our study has important strengths in informing the understanding of STI epidemiology in Zimbabwe. Use of RDS sampling ensured results are more representative of the network of FSWs in the community than convenience samples from clinic attendees. Limitations include partial sampling for STI testing—only one-third had testing and genital examination. Although randomly selected for invitation, some differences were found between those taking up and declining genital examination and STI testing. We are unable to report on individual HPV sub-types, instead grouping high-risk subtypes 18 to 45, and did not test for some other common STIs including bacterial vaginosis. Our data were collected in 2017, and it is possible that changes to sex work during the COVID-19 pandemic and any changes in use of HIV prevention products such as PrEP might have had effects on STI prevalence among FSWs; it is unlikely that these would change our observation of high prevalence or our observation that the screening algorithms we examined performed poorly with respect to sensitivity.

The prevalence of high-risk HPV, which is associated with cervical and vaginal cancers was high across the 3 sites, consistent with global meta-analyses of HPV prevalence among FSWs.^25^ Annual HPV vaccination using bivalent 2-dose vaccines (HPV genotypes 16 and 18) has been delivered to girls aged 10 to 14 years in Zimbabwe since 2018, with additional programs aimed at reaching out-of-school girls; in August 2019, 2-dose coverage of those eligible was estimated to be high at 75% to 86%.^13^ We have identified a wider range of high-risk HPV genotypes among this survey of FSWs, with 30% to 40% prevalence of genotypes other than types 16 or 18 of 45 among FSWs across sites. This range of genotypes is consistent with those identified among cervical cancer patients in Zimbabwe, suggesting that vaccines targeting a wider range of genotypes are required.^26^ Cervical cancer prevention that is tailored to the needs of current FSWs, most of whom are too old to have directly benefitted from the vaccination program, is critically needed.

Sexually transmitted infection declines documented in Zimbabwe in the 1990s and early 2000s have been attributed to increasing condom use as well as economic factors that reduced migrant labor and dampened demand for sex work.^8^ However, we found a high prevalence of STIs among FSWs across the 3 sites in our study, consistent with changes in sex work supply and demand occurring in Zimbabwe since the early 2000’s.^27^ The very high prevalence of gonorrhea, chlamydia, syphilis, trichomonas, and HPV reported here implies active transmission in sex work and low rates of consistent condom use, and highlights limitations of current clinical services to influence STI control. Such challenges have been recognized for decades in other countries and regions and remain significant barriers to reaching global STI elimination targets.^28^

Given these challenges, several options exist to interrupt STI transmission in sex work and reduce the prevalence of the STIs reported here: (1) effective STI services to detect and/or treat both symptomatic and asymptomatic STIs, and (2) condom programming that addresses structural barriers to consistent use in sex work.^27,29^ Because controlling transmission in the highest-risk sexual networks also reduces downstream transmission to lower-risk partners, investment in such interventions would contribute efficiently and cost-effectively to wider STI control.^30^

Currently in Zimbabwe, symptomatic STIs among FSWs are managed as they are for any woman, using syndromic guidelines. Screening for asymptomatic or inapparent STIs is limited to serological testing for syphilis. We explored several options for improving STI detection with screening algorithms adapted from national guidelines. About 10% more NG/CT could be detected with screening extended to women without symptoms (i.e., all sex workers), but nearly half the infections were still missed.

Our findings of poor sensitivity, specificity, and positive predictive values of vaginal discharge algorithms to detect gonorrhea and chlamydia infections are consistent with previously recognized limitations of this approach.^31s^ The overlapping symptoms of different causes of vaginal discharge, for example, discharge, itching, or odor, make it difficult to distinguish between them based on symptoms alone and can result in inappropriate treatment. The proportion of FSWs reporting vaginal symptoms who tested positive for chlamydia was 12.5% and for gonorrhea 18.9%, not dissimilar from an STI etiology survey conducted among sexual health clinic attendees in 2014/2015, which found that 14.0% of women reporting vaginal discharge tested positive for chlamydia and 24.0% for gonorrhea.^11^ Current syndromic algorithms simply fail to detect most infections; in this study, gonorrhea prevalence among sex workers presenting with symptoms was only marginally higher (16%) than among those without (9%). This detection gap represents the proportion of the population left untreated, which helps sustain high prevalence.

Routine screening is an alternative strategy that does not rely on symptoms, offering periodic checkups with affordable sensitive testing methods, underpinned by tailored approaches to engage FSWs in sexual health services, The Global HIV Prevention Coalition recommends quarterly medical checkups as part of key population “trusted access platforms” guidance informed by experience from Kenya, India, and elsewhere.^32s^ Resources remain a major barrier. Sufficiently sensitive diagnostic tests exist but remain unaffordable or impractical in many low-income countries. A rough estimate for quarterly nucleic acid amplification test screening (gonorrhea and chlamydia) is in the range of $100 per sex worker per year. This situation may change in the future if more affordable point-of-care tests with sufficiently high sensitivity become available. Recent expansion of rapid molecular diagnostic technology for tuberculosis and COVID-19 makes nucleic acid amplification test for NG/CT potentially more feasible if not more affordable, whereas improvements in lateral flow test technology may eventually offer simplicity and affordability. An important consideration for a feasible screening test is that results are available with little delay, so sex workers who test positive can be treated before leaving the clinic.

Epidemiologic treatment—based on high prevalence and risk of infection—is another option. Partner treatment may help identify some who need treatment, but in practice rarely identifies casual or commercial sex partners.^33s^ The current Zimbabwe STI case management guidelines include risk assessment—a partner with STI or a new partner in last 3 months—to guide treatment of NG/CT for symptomatic women. Virtually all sex workers meet one or both these criteria. By dropping symptoms as the algorithm entry point, which our findings show will miss many infections, all sex workers could be offered presumptive treatment at regular medical checkups based on risk, either once or on a periodic basis, if sensitive screening tests are not available. This approach has been effective in reducing STI prevalence in diverse settings.^34s^ Doxycycline postexposure prophylaxis could potentially have been another intervention to prevent bacterial STIs after condomless sex, but a recent study in Kenya found no evidence that the incidence differed for chlamydia, syphilis, or gonorrhea among women assigned to doxycycline postexposure prophylaxis and those who were not.^35s^

The combination of revived and enhanced condom programming, regular checkups with more effective STI screening, and consideration of presumptive antibiotic treatment or, if found to be effective, antibiotic postexposure prophylaxis, together with HIV-specific prevention and treatment and cervical cancer prevention, offers the potential for balanced and comprehensive prevention and control of the full gamut of STIs. Whatever combination of screening and epidemiologic treatment is used, effective STI services are essential for FSW in Zimbabwe, and it is critical to plan and implement such services in partnership with sex workers.

## Appendix

For further references, please see “Supplemental References,” http://links.lww.com/OLQ/B143.
